# Parasite-Derived Proteins for the Treatment of Allergies and Autoimmune Diseases

**DOI:** 10.3389/fmicb.2017.02164

**Published:** 2017-11-07

**Authors:** Zhenyu Wu, Lifu Wang, Yanlai Tang, Xi Sun

**Affiliations:** ^1^Department of Parasitology, Zhongshan School of Medicine, Sun Yat-sen University, Guangzhou, China; ^2^Key Laboratory of Tropical Disease Control (SYSU), Ministry of Education, Guangzhou, China; ^3^Provincial Engineering Technology Research Center for Diseases-Vectors Control, Guangzhou, China; ^4^Department of Pediatrics, The First Affiliated Hospital, Sun Yat-sen University, Guangzhou, China

**Keywords:** parasite, product, hygiene hypothesis, autoimmune disease, allergy

## Abstract

The morbidity associated with atopic diseases and immune dysregulation disorders such as asthma, food allergies, multiple sclerosis, atopic dermatitis, type 1 diabetes mellitus, and inflammatory bowel disease has been increasing all around the world over the past few decades. Although the roles of non-biological environmental factors and genetic factors in the etiopathology have been particularly emphasized, they do not fully explain the increase; for example, genetic factors in a population change very gradually. Epidemiological investigation has revealed that the increase also parallels a decrease in infectious diseases, especially parasitic infections. Thus, the reduced prevalence of parasitic infections may be another important reason for immune dysregulation. Parasites have co-evolved with the human immune system for a long time. Some parasite-derived immune-evasion molecules have been verified to reduce the incidence and harmfulness of atopic diseases in humans by modulating the immune response. More importantly, some parasite-derived products have been shown to inhibit the progression of inflammatory diseases and consequently alleviate their symptoms. Thus, parasites, and especially their products, may have potential applications in the treatment of autoimmune diseases. In this review, the potential of parasite-derived products and their analogs for use in the treatment of atopic diseases and immune dysregulation is summarized.

## Introduction

Parasites, which have co-evolved with mammals, including humans, over a long period of time, can use various strategies to survive, such as secreting immune-evasion molecules to modulate the host immune system. However, consequently, parasites play a dual role in human health (Ditgen et al., 2014; Maizels and McSorley, 2016). On the one hand, parasites threaten human health by causing malnutrition, mechanical damage, and toxic effects. On the other hand, parasites induce potent immunomodulatory effects and can act as potential therapeutics for a variety of inflammatory diseases including asthma, rhinitis, intestinal inflammation disease, type 1 diabetes (T1D), and several other immune dysregulation disorders.

Conventional wisdom suggests that there are three consequences of parasitic infection: (1) The damage caused by the host response exceeds the ability of the parasites to adapt, the parasites are all cleared, and the host can resist or partially resist re-infection; (2) the host can only remove some of the parasites, the infection becomes chronic so that the host is a parasite carrier, and the host has partial resistance against re-infection (most host–parasite interactions belong in this category); (3) the parasites cause many pathogenic reactions or high levels of pathogenicity and the host cannot effectively remove the parasites, leading to significant pathological changes and clinical symptoms.

Parasitosis is a major public health problem worldwide and seriously threatens human health and life, especially in underdeveloped areas (Cooke, 2009; de Ruiter et al., 2017). According to the World Health Organization, more than a billion people around the world suffer from parasitic diseases. Thus, parasites, as enemies of human health, cause serious damage to human health. The activities of parasites, including invasion, migration, settlement, development and breeding, lead to damage to the host cells, tissues and organs, and even to the whole system.

However, with the decrease in parasitic infections, the incidence and harmfulness of atopic diseases (including asthma, allergic rhinitis, food allergies, anaphylaxis, and atopic dermatitis) and immune-mediated disorders (including T1D and inflammatory bowel disease) have been increasing remarkably all over the world, especially in developed countries (Heylen et al., 2014b; Stiemsma et al., 2015). Greenwood (1969) first observed the negative correlation between parasites and immune dysregulation. The low incidence of rheumatoid arthritis in Western Nigeria may be related to exposure to the malaria parasite (Greenwood, 1969; Garg et al., 2014). Later, Greenwood et al. (1970) observed that infection by *Plasmodium berghei* can suppress spontaneous autoimmune disease in mice. The “hygiene hypothesis” was then proposed by Strachan (1989). According to this hypothesis, exposure to infectious diseases in childhood can prevent adults from developing atopic diseases. As a result, the increase in atopic diseases can be attributed to the control of infectious agents such as parasites, especially helminths (Strachan, 1989).

Helminth infections mainly occur in developing countries and they are rare in industrialized counties. Helminths can be divided into two phyla: Platyhelminthes (flat worms), including both trematodes and cestodes, and Nemathelminths, including nematodes. They are mostly found in the gastrointestinal tract. Helminths have co-evolved with their hosts, such as humans, for millions of years. They can parasitize their hosts for a long time. For example, *Wuchereria bancrofti* can live in the human body for more than 10 years and *Trichinella spiralis* for up to 30 years (Harnett and Harnett, 2010). The goal of the parasitism is not to kill the host but to survive for as long as possible by maintaining a state of balance with the host. Thus, parasites need to employ various mechanisms to modulate the host immune system in order to prevent activation that may lead to their elimination and, at the same time, not cause serious immunosuppression that leads to the death of the host induced by a subsequent infectious disease. This mechanism of immunomodulation, avoiding excessive activation of the host immune system, can also be used by the host to protect against certain inflammatory disorders.

According to the hygiene hypothesis, humans have established an immunological balance between the TH1/TH17 and TH2 responses, of which the TH1/TH17 response is related to bacterial infections and autoimmunity and the TH2 response is related to parasite infections and allergies. Parasite infection stimulates a strong TH2 response but inhibits the TH1 response, resulting in the inhibition of autoimmunity. Based on this finding, it seems that parasites can hinder the progression of allergic disease. In fact, parasite infection cannot induce the formation of allergy-related IgE but it can induce the formation of polyclonal IgE, which is not capable of inducing allergic diseases (Erb, 2009). In addition, parasites can induce immune tolerance by secreting anti-inflammatory products to modulate the immune response (Yazdanbakhsh et al., 2002; Li et al., 2017). Furthermore, parasites can also amplify T-regulatory (Treg) and B-regulatory (B-reg) cells and type-2 macrophages, hamper dendritic cells (DCs) toward the tolerogenic phenotype, downregulate type-2 innate lymphoid cells (ILC2), and further modulate the gut microbiota (Rook, 2012; Versini et al., 2015).

Many attempts have been made to administer parasites, especially helminths, to treat allergies and autoimmunity. Different species of helminths and administration approaches have been evaluated in experimental models and clinical studies (**Table 1**). In a murine model of experimental autoimmune encephalomyelitis (EAE), the injection of schistosome eggs relieved the symptoms and prevented relapse (Zheng et al., 2008). However, the direct application of parasites is less likely to be acceptable to patients as they may develop parasitic diseases. Considering that parasite-derived antigens may have similar functions to the parasite itself, some researchers have attempted to use parasite-derived antigens instead of the parasite itself to obtain desirable outcomes. van Die and Cummings (2010) discovered that parasitic proteins such as surface and secretory antigens can inhibit the immune response, so they represent potential treatments for atopic diseases.

**Table 1:** Experimental studies and human trials of parasite-derived therapies for important autoimmune diseases.

Autoimmune disease	Parasite	Effect	Useful component	Reference
Inflammatory bowel disease (IBD)	*Schistosoma mansoni*	S. mansoni cercaria infection attenuates DSS-induced colitis in mice. Immunization with P28GST, a unique recombinant schistosome enzyme, ameliorates intestinal inflammation by eosinophil-dependent modulation of harmful type 1 responses, representing a new immunoregulatory strategy against IBD (Driss et al., 2016).	Soluble egg antigen (SEA), soluble worm antigen (SWA), glutathione *S*-transferase P28GST, Sj16	Ruyssers et al., 2009; Bodammer et al., 2011; Heylen et al., 2014a; Hasby et al., 2015; Driss et al., 2016
	*Schistosoma japonicum*	*S. japonicum* ova pre-treatment contributes to the relief of colitis and decreases mortality in a TNBS-induced colitis model, modulating the activity of CD4+CD25+Treg cells.	Cystatin	Xia et al., 2011; Wang et al., 2016
	*Ancylostoma caninum*	Ameliorates TNBS-induced colitis in mice, decreases production of pro-inflammatory cytokines (IFN-γ and IL-17), and increases production of regulatory cytokines (IL-10 and TGF-β) in colon tissue.	Soluble antigen	Ruyssers et al., 2009
	*Hymenolepis diminuta*	Colitis suppressed by adoptive transfer of antigen-treated dendritic cells.	Adult worm extract	Reyes et al., 2015, 2016a; Matisz et al., 2017
	*Anisakis*	*Anisakis* excretory-secretory (ES) compounds have a protective effect on intestinal immunopathology.	ES products isolated from *Anisakis* larvae	Haarder et al., 2017
	*Ascaris lumbricoides*	Reduces inflammation in a mouse model of DSS-induced colitis, probably by increasing the expression of anti-inflammatory cytokines (IL-10 and TGF-β) and reducing pro-inflammatory ones (IL-6 and TNF-α).	Recombinant cystatin	Coronado et al., 2017
	*Heligmosomoides polygyrus*	Alteration of the gut microbiota is a significant contributor to the *H. polygyrus*-induced exacerbation of *C. rodentium* colitis.		Su et al., 2017
	*Trichuris suis*	Clinical trials using *T. suis* for treatment of active Crohn’s disease and ulcerative colitis showed that most of the patients experienced clinical improvement and no adverse effects.	Ova	Summers et al., 2003, 2005a,b; Elliott and Weinstock, 2017; Su et al., 2017;
	*Necator americanus*	Clinical trials showed that *N. americanus* L3 may have a therapeutic effect on Crohn’s disease, with improved CDAI in 5/9 patients at 20 weeks and 3/5 patients at 45 weeks, and mild adverse events.		Elliott and Weinstock, 2017
	*Clonorchis sinensis*	Reduces DSS-induced colitis by induction of IL-10 production by macrophages.	Recombinant type-1 cystatin (Csstefin-1)	Jang et al., 2011
	*Brugia malayi*	Reduces T-cell-mediated colitis by reducing IFNr and IL-17 in the spleen, mesenteric lymph nodes, and lamina propria, and increasing IL-4 and IL-10.	Recombinant asparaginyl-tRNA synthetase (AsnRS)	Kron et al., 2013
	*Echinococcus granulosus*	Attenuates DSS-induced colitis by reducing iNOS expression in the colon but not in the plasma	Laminated layer crude extract	Soufli et al., 2015
Rheumatoid arthritis	*Schistosoma mansoni*	Protective effects on the clinical and immunopathological features of rheumatoid arthritis by reducing serum IL-17 and increasing IFN-r and IL-10. Reduces collagen-induced arthritis by downregulation of the Th1 (IFN-r) response and pro-inflammatory cytokines (TNF-α and IL-17A), and upregulation of the Th2 (IL-4) response and an anti-inflammatory cytokine (IL-10).	Autoclaved *S. mansoni* antigen (ASMA)	Eissa et al., 2016
	*Schistosoma japonicum*	Attenuates murine collagen-induced arthritis by augmenting IL-4, IL-10, and collagen-specific IgG1, distinctly reducing IFN-r and collagen-specific IgG2a, and decreasing IL-6, IL-17, TNF-α, and RANKL. Reduces severity of complete Freund’s adjuvant-induced arthritis in a rat model by increasing production of IL-10 and IL-4 and decreasing production of IL-12p70 and IFN-r.	Recombinant SjCystatin and Sj16	Sun et al., 2010; Song et al., 2011; Liu et al., 2016
	*Schistosoma japonicum*	Attenuates murine collagen-induced arthritis by augmenting IL-4, IL-10, and collagen-specific IgG1, distinctly reducing IFN-r and collagen-specific IgG2a, and decreasing IL-6, IL-17, TNF-α, and RANKL. Reduces severity of complete Freund’s adjuvant-induced arthritis in a rat model by increasing production of IL-10 and IL-4 and decreasing production of IL-12p70 and IFN-r.	Recombinant SjCystatin and Sj16	Sun et al., 2010; Song et al., 2011; Liu et al., 2016
	*Trichinella spiralis*	Protective effects on the clinical and immunopathological features of rheumatoid arthritis by reducing serum IL-17 and increasing IFN-r and IL-10.	Autoclaved *T. spiralis* antigen (ATSA)	Eissa et al., 2016
	*Fasciola hepatica*	Inhibits the symptoms of collagen-induced arthritis through Foxp3^+^ regulatory T cells	*F. hepatica* total extract	Carranza et al., 2012
	*Hymenoleptis diminuta*	Attenuates complete Freund’s adjuvant-induced arthritis. Protection abrogated in mice lacking T and B cells or IL-4Ra or IL-10.		Shi et al., 2011
Multiple sclerosis	*Schistosoma mansoni*	Alters the progression of EAE. Increases IL-4, IL-10, and TGF-β, decreases IFN-r, TNF-α, and IL-12, and changes CNS inflammation.	Ova, soluble egg antigens (SEA)	La Flamme et al., 2003; Sewell et al., 2003; Zheng et al., 2012
	*Trichuris suis*	Reduces disease severity in a multiple sclerosis model. Markedly reduces Th1 and Th17 responses.	ES products	Hansen et al., 2017
	*Toxascaris leonina*	Inhibits remission of EAE, markedly increases the number of CD45R/B220(+)B cells, and decreases INF-r and TNF-α.	Recombinant galectin (rTl-gal) isolated from adult worms	Bing et al., 2015
	*Trichinella spiralis*	Ameliorates EAE in mice and DA rats. Shifts the Th2 response and increases the number of Treg cells.	ES product from muscle L1 larvae (ESL1)	Gruden-Movsesijan et al., 2008, 2010; Radovic et al., 2015
	*Schistosoma japonicum*	Attenuates EAE. Increases IL-4 and decreases IFN-r and CNS inflammation.	Soluble egg antigen (SEA)	Zheng et al., 2008
	*Fasciola hepatica*	Attenuates EAE. Enhances anti-inflammatory cytokines (IL-33 and IL-5) and Th2 responses. Protection against EAE is independent of IL-4, IL-10, and Treg cells.	ES products (FHES)	Finlay et al., 2016
	*Taenia crassiceps*	Suppresses EAE development. Induces a range of Th2-type cytokines, while suppressing Th1 and Th17 responses, and increases tolerogenic DC and M macrophages.	ES products (TcES)	Finlay et al., 2016
	*Plasmodium chabaudi*	Modifies the clinical course of EAE. Decreases IL-17 and IFN-r, and increases IL-10, TGF-β1, and Treg cells.		Farias et al., 2011
Systemic lupus erythematosus (SLE)	*Plasmodium chabaudi*	Ameliorates the histopathological changes in a murine model of SLE. Significantly increases plasma levels of IL-4, IL-6, IL-7, IL-12, IL-17, IFN-α, IFN-γ, TGF-β, BAFF, and APRIL and markedly increases IgG2a, IgG3, and anti-dsDNA autoantibodies. Attenuates B cell autoreactivity by modulating the CXCL12/CXCR4 axis and its downstream signals PI3K/AKT, NFκB, and Erk.		Badr et al., 2015; Abdel-Maksoud et al., 2016
	*Acanthocheilonema viteae*	Protective effect on SLE by downregulating myeloid differentiation factor 88 (MyD88) expression by B cells and kidney cells.	ES-62	Rodgers et al., 2015
	*Schistosoma mansoni*	Modifies the cytokine microenvironment and alters the pathological phenotype of autoimmune nephritis. Skews the immune response to the Th2 response and increases IL-10.		Miyake et al., 2014; Savio and Coutinho-Silva, 2016
Type 1 diabetes	*Schistosoma mansoni*	Ameliorates streptozotocin-induced T1D in wild-type mice via a Treg/IL-4/IL-13/IL-10-independent mechanism that may be related to augmented expression of Arg-1 and Ym1.		Savio and Coutinho-Silva, 2016
	*Brugia malayi*	rBMALT-2 has effective immunomodulatory and anti-diabetic effects by skewing the immune response to the Th2 response.	Filarial abundant larval transcript 2 (ALT-2)	Reddy et al., 2017
	*Fasciola hepatica*	Ameliorates SLE and modulates macrophage function.	FhHDM-1	Reddy et al., 2017
	*Trichinella spiralis*	Prevents diabetes in NOD mice by disrupting the pathways leading to the Th1-mediated destruction of insulin-producing beta cells.		Saunders et al., 2007
	*Dirofilaria immitis*	Prevents diabetes in NOD mice and increases IgE.		Imai et al., 2001

Thus, in this review, we will summarize various parasite-derived products and their mechanisms to protect against atopic and autoimmune diseases.

## Es-62

*Acanthocheilonema viteae* has been reported to live for a long time in humans, and ES-62 is a major excretory-secretory (ES) product of *A. viteae* that has been shown to modulate the human immune response in an early time (Stepek et al., 2002; Houston and Harnett, 2004). ES-62 is a glycoprotein with a phosphorylcholine (PC) attached to the uncharacterized *N*-glycan, and PC plays a central role in immune regulation (Agrawal et al., 2003).

ES-62 can promote a TH2 response via an interaction with Toll-like receptor 4 (TLR4). Normal TLR4 can recognize pathogen-associated molecular patterns (PAMPs) such as lipopolysaccharide (LPS) to recruit myeloid differentiation primary-response protein 88 (MyD88), which induces the phosphorylation and activation of mitogen-activated protein kinases (MAPKs) such as p38 MAPK, Erk and JNK. In addition, the recruitment of MyD88 can activate the nuclear factor (NF)-κB signaling pathway. Both the activation of MAPK kinases and NF-κB signaling result in the production of pro-inflammatory molecules such as interleukin (IL)-6 and IL-12 (Melendez et al., 2007). Nevertheless, ES-62 is able to inhibit the JNK pathway and p38 MAPK pathway but stimulate the sustained activation of Erk, and Erk can inhibit the production of IL-12 via the transcription factor FOS (Lal et al., 1987; Goodridge et al., 2005b). Moreover, ES-62 can induce transient activation of the NF-κB signaling pathway, which can induce a transformation from pro-inflammatory activity to an anti-inflammatory TH2 response (Goodridge et al., 2005b; Kawai and Akira, 2010). Furthermore, the inhibition of JNK kinase by ES-62 can hinder the production of p35 subunit, the component of pro-inflammatory cytokines IL-6, IL-10, and TNF (Goodridge et al., 2005a).

ES-62 can modulate the activation of lymphocytes through the modulation of BCR and TCR. Normally, immunoreceptor tyrosine activation motifs (ITAMs) in BCR can be phosphorylated to induce the activation of the phosphoinositide 3-kinase (PI3K) and PLCγ signaling pathways, both of which can induce the activation of PKCs such as aPKC, cPKC, and nPKC. In addition, phosphorylated ITAMs can recruit the Grb2/Sos complex, leading to the activation of Erk (Thomas et al., 2003). However, ES-62 can activate the SHP-1 tyrosine phosphatase to dephosphorylate and inactivate the ITAMs and consequently modulate the signaling pathways. Moreover, RasGAP and Pac-1 are recruited and inhibit Ras and Erk, respectively. ES-62 can also induce the degradation of PKCs to further modulate the PI3K and PLCγ signaling pathway (Goodridge et al., 2007). Similarly, the phosphorylation of ITAMs in TCR can lead to the recruitment of PTKs and the subsequent activation of the PI3K and PLCγ signaling pathway, both of which can induce the activation of PKCs (Deehan et al., 1998). ES-62 can modulate the PI3K and PLCγ signaling pathways, but not the activation of PTKs, to promote the anti-inflammatory activity (Deehan et al., 2001).

ES-62 can also block mast cell degranulation, leading to inhibition of the release of pro-inflammatory cytokines such as prostaglandins and leukotrienes. Normally, an allergen or an allergen binding to Fc𝜀RI and recruiting PKCs will lead to the activation of the PLD/SPHK1/calcium signaling pathway. As a result, the level of calcium is increased, which induces the degranulation of the mast cells and the initiation of an allergic response. In contrast, ES-62 binds to TLR4 and leads to the sequestration and degradation of PKCα, resulting in the uncoupling of the PLD/SPHK1/calcium signaling pathway. The level of calcium is decreased, which consequently suppresses the degranulation of mast cells (Harnett et al., 1998).

In addition, recent studies have demonstrated that ES-62 can also modulate complement activation. In the serum of individuals infected with *A. viteae*, ES-62 can bind to C-reactive protein (CRP) to form an ES-62-CRP-C1 complex (Marshall et al., 2005; McGrath et al., 2006). The flexible nature of PC attached to CRP leads to the uncoupling of C1s and C4b, resulting in a failure of C2 cleavage. Moreover, ES-62 may be associated with the degradation of early complement components such as C4, which can also help suppress complement activation (Ahmed et al., 2016).

Animal experiments have also confirmed that ES-62 has a protective effect against some autoimmune diseases. The parasitic worm ES product has a protective effect against disease in a mouse collagen-induced arthritis model of rheumatoid arthritis (RA) by suppressing pathogenic IL-17 and upregulating IL-22 production by recruiting γδ T cells (Pineda et al., 2014, 2015).

ES-62 can also reduce lupus-associated accelerated atherosclerosis in a mouse model. Although treatment with ES-62 cannot substantially modulate renal pathology, it can decrease anti-nuclear autoantibody levels, causing a 60% reduction in aortic atherosclerotic lesions and an associated decrease in macrophages and fibrosis (Aprahamian et al., 2015). ES-62 also protects against ovalbumin-induced airway hyper-responsiveness in mice as a result of the covalently attached anti-inflammatory PC residues. Thus, PC plays a central role in the anti-inflammatory activity of ES-62 (Harnett and Harnett, 2010).

A library of 79 small-molecule analogs (SMAs) of ES-62 based around PC have been screened, and four synthetic ES-62-related SMAs (SMAs-11a, 11e, 11i, and 12b) were found to have similar modulatory effects to ES-62 and no immunogenicity. Sulfore-type SMAs can modulate PAMP-mediated pro-inflammatory cytokine production and drive Th1/Th17 responses. In addition, SMAs can protect against allergies and some autoimmune diseases. For example, two SMAs, sulfone compounds termed 11a and 12b, which are the major SMAs that have been discovered, have been shown to protect against clinically relevant allergens. The SMAs of ES-62 based around its active PC moiety also offer some protection against the development of pulmonary allergic responses to allergens (Janicova et al., 2016). Transferring DCs treated with SMAs to mice can significantly reduce the severity of collagen-induced arthritis, which is accompanied by a significant generation of Foxp3^+^CD4^+^ Treg cells and/or the production of IL-10, and a reduction in IL-17^+^ cells in the draining lymph nodes (Lumb et al., 2017).

## *Fasciola hepatica* Excretory-Secretory Products (FHES, FhCL1, and FhHDM)

*Fasciola hepatica* excretory-secretory was found to be useful for diagnosing human fascioliasis (Haseeb et al., 2003). Using gel filtration chromatography, FHES can be divided into two components, FhCL1 and FhHDM. FhCL1 is a kind of cathepsin L cysteine protease and FhHDM is a peptide with a cathelicidin-like C-terminal alpha helix (Robinson et al., 2009, 2011).

*Fasciola hepatica* excretory-secretory has been demonstrated to protect against T1D in non-obese diabetic (NOD) mice (Lund et al., 2014). In NOD mice, there is infiltration of anti-autoantigen CD4^+^ T cells into the pancreatic lymph nodes (PLNs), which causes immune-system-mediated injury of the pancreatic islets (Jaakkola et al., 2003). In contrast, after administering FHES, plenty of IL-10-producing B cells rather than CD4^+^ T cells can be observed in PLNs, inhibiting the development of autoimmune diabetes in an IL-10-dependent manner. In addition, in NOD mice, an increased number of M1 macrophages are able to secrete IL-12, promoting auto-antigen processing and a subsequent autoimmune response (Alleva et al., 2000). However, FHES can increase the level of regulatory M2 macrophages with increased Ym1, TGF-β, Arg-1, and PD-L1 expression, leading to modulation of the immune reaction in the pancreatic islets. In addition, M2 macrophages can induce the activation of CD4^+^ CD25^+^ Foxp3 Treg cells in a TGF-β-dependent manner (Lund et al., 2014).

Furthermore, FHES has been shown to protect against EAE in a murine model. FHES can enhance the TH2 response and reduce the TH1/TH17 response, which consequently reduces the TH1 and TH17 cell infiltration into the central nervous system (CNS) (Finlay et al., 2016). Moreover, injecting FHES can increase anti-inflammatory cytokine levels, but the function of FHES is independent of anti-inflammatory cytokines such as IL-4 and IL-10. In contrast, the improvement of EAE symptoms depends on an increase in type 2 cytokines such as IL-5 and IL-33, which can induce the accumulation of eosinophils (Finlay et al., 2016). Previous research has demonstrated that the differentiation, maturation, and infiltration of eosinophils, which have immunomodulatory functions, are dependent on IL-5 (Ben Baruch-Morgenstern et al., 2014). As a result, after the injection of FHES, there is eosinophil infiltration into the CNS, which helps to alleviate the immune response associated with EAE. In addition, IL-33 can improve the clinical symptoms of EAE and increase eosinophil infiltration and, after FHES administration to IL-33^-/-^ mice, protection against EAE is changed, although the underlying mechanism remains elusive (Finlay et al., 2016).

Researchers have isolated FhCL1 and FhHDM to study their immunomodulatory effects. FhCL1 has been shown to suppress the production of IFN-γ. The protective effect of FheCL is associated with IL-4 because FheCL cannot function in an IL-4-defective murine model (O’Neill et al., 2001). Further study has shown that FheCL can inhibit the secretion of pro-inflammatory cytokines such as nitric oxide, IL-6, IL-12, and IFN-γ. The decreased level of pro-inflammatory cytokines is dependent on the inhibition of TLR3 and TLR4 via the suppression of the TRIF-dependent signaling pathway but not the MyD88-dependent pathway (Coakley et al., 2016). Interestingly, the inhibition of the pro-inflammatory signaling pathway depends on TLR3 degradation in endosomes, but why TLR3 degradation can affect the TLR4 signaling pathway is still unclear. The inhibition of nitric oxide, IL-6, IL-12, and IFN-γ caused by FheCL has been taken advantage of to relieve the symptoms of endotoxic shock (Coakley et al., 2016).

As another component of FHES, FhHDM has been discovered to protect against atopic diseases (Alvarado et al., 2015). In a recent study, FhHDM was shown to alleviate the symptoms of T1D and EAE. However, FhHDM cannot inhibit the TH1 response or induce the TH2 response to modulate the immune response. The differences between FhHDM-1- and PBS-treated mice in the response to auto-antigens and in the production of cytokines by T cells are not significant (Lund et al., 2016). In addition, after the administration of FhHDM, there is no significant difference in the levels of pro-inflammatory cytokines, such as IFN-γ, IL-17, and TNF, and anti-inflammatory cytokines, such as IL-4 and IL-10. In contrast, FhHDM can inhibit the secretion of pro-inflammatory cytokines, such as TNF and IL-6, by macrophages in a dose-dependent manner instead of modulating the expression of co-stimulatory signaling pathways. According to previous research, although CD4^+^ T cells play a key role in the progression of many autoimmune diseases such as T1D and EAE, the pro-inflammatory cytokines secreted by macrophages are involved in the initiation and progression of autoimmune disease via an interaction with CD4^+^ T cells (Jun et al., 1999). Thus, FhHDM protects against autoimmune diseases such as T1D and EAE via the inhibition of pro-inflammatory cytokine-producing macrophages, instead of altering the macrophage phenotype or directly modulating the T cell function.

## *Hymenolepis diminuta* Antigen (HdAg)

*Hymenolepis diminuta* infection has been discovered to ameliorate the symptoms of dextran sodium sulfate (DSS)-induced colitis, which is a model of human inflammatory bowel disease (Reardon et al., 2001). HdAg has been shown to relieve DSS-induced colitis, with decreased expression of IFN-γ, IL-17, and TNF and increased expression of IL-10 (Reyes et al., 2016b). In a murine model of DSS-induced colitis, the administration of HdAg increases the level of CCR2-dependent immunoregulatory F4/80^+^ Gr-1^lo^ LyC6^+^ monocyte-like cells in the peritoneal cavity. These cells, which can express programmed death ligand 1 (PD-L1), cannot modulate the proliferation of T cells directly but can enhance the production of IL-10 and IL-4 to suppress the immune response, which is a characteristic of myeloid-derived suppressor cells (MDSCs). However, whether the cells are MDSC or not is still unknown. In addition, the adoptive transfer of monocyte-like cells to HdAg-free mice can also inhibit the progression of DSS-induced colitis (Reyes et al., 2016b).

Matisz et al. (2015) tested whether DCs pulsed with HdAg can ameliorate the severity of hapten-induced colitis, just as direct HdAg administration can. The research has demonstrated that the administration of *H. diminuta*-pulsed (HD)-DCs can significantly ameliorate the severity and reduce the mortality of DSS-induced colitis, with an enhanced TH2 response and expression of TH2 cytokines such as IL-10, which is produced mainly by CD4^+^ T cells. The anti-colitis function of IL-10 appears to be a key, because there is no protection against colitis when HD-DCs are transferred into IL-10 knockout mice. In addition, further research has investigated the detailed mechanism of HD-DC function and demonstrated the importance of both IL-4 and IL-10 (Matisz et al., 2017). HD-DCs can produce IL-10, which induces recipient cells to secrete IL-4 and IL-10, and the IL-4 can then activate both the recipient cells and HD-DCs; HD-DCs cannot directly produce IL-4. Both IL-4 and IL-10 have anti-colitic action.

## *Trichinella spiralis* Excretory-Secretory Muscle Larvae (ES L1)

Prior infection with *T. spiralis* in a murine model of hapten-induced colitis has been shown to ameliorate the clinical symptoms of colitis (Khan et al., 2002). Motomura et al. (2009) extracted ES L1 to show that prior administration of the antigen can ameliorate the severity and reduce the mortality of hapten-induced colitis in a murine model. In their research, ES L1 was extracted from *T. spiralis* muscle L1 larvae. Prior submucosal application of the antigen enhances the TH2 response as well as the Treg response and reduces the TH1 response. The TH2 response can induce the production of IL-13 and the Treg response can induce the production of TGF-β, both of which help to modulate the aberrant immune response (Motomura et al., 2009).

Further examination has revealed that the administration of the antigen can lower the activity of myeloperoxidase (MPO) and the expression of IL-1β and iNOS. Previous research has demonstrated that MPO is located in various inflammatory cells such as neutrophils and it is regarded as a marker of inflammation (Smith and Castro, 1978). A decrease in MPO activity indicates a diminished immune response. IL-1β and iNOS play an important role in the initiation of inflammation, and downregulation indicates a lowered immune response. Further research has shown that prior administration of ES L1 can ameliorate EAE in a murine model, in a process in which CD4^+^ CD25^-^ Foxp3^+^ regulatory cells play an important role (Radovic et al., 2015).

## Autoclaved *Schistosoma mansoni* Antigen (ASMA) And Autoclaved *Trichinella spiralis* Antigen (ATSA)

Researchers tested the protective effects of the administration of ASMA and ATSA in a rat model of adjuvant-induced arthritis (AA), which is similar to human RA (Eissa et al., 2016). The intradermal injection of ASMA or ATSA can ameliorate the clinical symptoms of AA with a significant increase in Foxp3^+^ Treg cells, and elevated levels of IL-17, IFN-γ, and IL-10. IL-17 and IL-10 can inhibit the progression of AA, but IFN-γ normally promotes an inflammatory response, which makes these findings controversial. However, a previous study showed that IFN-γ may have an anti-inflammatory effect in certain situations, although the detailed mechanism has yet to be determined (Krakowski and Owens, 1996). Thus, the increase in IFN-γ after treatment with ASMA or ATSA may have a protective effect on AA (Matthys et al., 1999).

## Lacto-N-Fucopentaose (LNFPIII)

Lacto-*N*-fucopentaose is an antigen with a Lexis trisaccharide that is secreted by *S. mansoni*. Similar to ES-62, LNFPIII can induce a TH2 response via the sustained activation of Erk, transient activation of the NF-κB signaling pathway, and inhibition of the JNK and p38 MAPK pathways (Thomas et al., 2003). Moreover, LNFPIII can interact with APCs through C-type receptors such as DC-specific ICAM3-grabbing non-integrin (DC-SIGN) to induce anti-inflammatory activation (Van Liempt et al., 2004). Further research has shown that the interaction between LNFPIII and TLR4 is dependent on CD14 with clathrin to induce endocytosis of the complex (Srivastava et al., 2014; Tundup et al., 2015). In addition, LNFPIII can induce the production of IL-10 by B1 cells, which may modulate the alternative activation of macrophages. LNFPIII has been demonstrated to protect against hepatosteatosis, T1D, and EAE (Bhargava et al., 2012; Zhu et al., 2012).

## *S. mansoni* Soluble Egg Antigen (SEA)

*Schistosoma mansoni* SEA has two components, IPSE and Omega-1. IPSE can trigger basophils to produce IL-4 and IL-13 via an interaction with IgE, leading to TH2 polarization (Li et al., 2013). Structural analysis has shown that IPSE is a member of the βγ-crystallin superfamily that can interact with IgE via crystalline folds in a crosslink-independent pathway (Meyer et al., 2015). Another component of SEA, Omega-1, also has a protective effect against atopic diseases, which depends on its glycosylation and RNase activity. The glycan of Omega-1 can bind to the mannose receptor on DCs and mediate the internalization of SEA, while the RNase activity is associated with the degradation of ribosomal and messenger RNA, leading to the inhibition of protein synthesis (Eissa et al., 2016). It is thought that protein synthesis inhibition can induce TH2 polarization via an unknown mechanism. Furthermore, Omega-1 can induce the secretion of IL-1β by macrophages (which are stimulated by TLR2) via an interaction with the C-type lectin receptor Dectin-1 (Everts et al., 2012). The activation of Dectin-1 leads to the formation of a caspase-8 inflammasome, which mediates the cleavage of pro-IL-1β, leading to an increase in IL-1β secretion. In addition, further study has shown that omega-1 can induce the production of Foxp3^+^ Treg cells (Ferguson et al., 2015).

## *Dirofilaria immitis* Derived-Antigen Product (DiAg)

DiAg, a *D. immitis*-derived product, can induce the production of IL-4 and IL-10, leading to the inhibition of the TH1 response as well as IgG production in a murine model (Imai and Fujita, 2004). Moreover, with the help of IL-4, DiAg can induce the production of polyclonal IgE via interacting with CD40 on the surface of B cells. In addition, DiAg can trigger the production of antigen-non-specific IgE, which can saturate Fc𝜀RI on the surface of mast cells. DiAg has also been discovered to relieve the symptoms of T1D in a murine model (Imai and Fujita, 2004).

## *Schistosoma mansoni* Derived Chemokine Binding Protein (smCKBP)

*Schistosoma mansoni* derived chemokine binding protein is a special protein that can interact with specific chemokines and prevent them from binding to and activating their receptors, leading to the inhibition of inflammatory cell activation and migration. smCKBP can interact with CXC-chemokine ligand 8 (CXCL8), CX3C-chemokine ligand 1 (CX3CL1), CC-chemokine ligand 3 (CCL3), CCL2, and CCL5, of which CCL2 and CCL5 can regulate the activation of normal T cells. In particular, the interaction and inhibition of CXCL8 can prevent the migration and infiltration of neutrophils in a murine model of air pouch inflammation (Tundup et al., 2015).

## Cystatin

Cystatin is a cysteine protease inhibitor that can be secreted by many parasites such as *A. viteae* and *Onchocerca volvulus*. Cystatin can induce IL-10-producing macrophages, resulting in a decreased expression of APC co-stimulatory molecules such as CD86 (van der Kleij et al., 2002). The interference with antigen processing leads to the inhibition of T cell activation. Another cystatin derived from *S. japonicum* has been found to inhibit the activation of lysosomal cysteine protease, leading to the inhibition of antigen presentation by DCs. In addition, the activation of CD4^+^ CD25^+^ Foxp3^+^ T cells and the expression of IL-4 and TGF-β are significantly enhanced after the administration of cystatin (Chen et al., 2017). Cystatin has been found to relieve the symptoms of ovalbumin-induced airway hypersensitivity and DSS-induced colitis (Hartmann and Lucius, 2003; Schnoeller et al., 2008).

## Sj16

Sj16 is a *S. japonicum*-derived protein (with a molecular weight of 16 kDa) that can be produced and secreted by all stages of *S. japonicum*. Research has confirmed that Sj16 is an important protein in the alleviation of inflammatory damage associated with cercariae penetration into the skin, and that it is intimately involved in the immune escape of *S. japonicum* (Hu et al., 2009). Recombinant Sj16, produced by *Escherichia coli*, has a definite anti-inflammatory effect both *in vivo* and *in vitro*. rSj16 has been shown to dramatically suppress the thioglycolate-mediated recruitment of leukocytes to the peritoneal cavity of BALB/c mice by upregulating IL-10 and IL-1Ra transcripts and downregulating IL-12p35, IL-1β, and MIP-2 transcripts (Hu et al., 2012). Additionally, rSj16 has a potent immunomodulatory effect and it was found to significantly alleviate AA in a rat model (Sun et al., 2010). rSj16 was also reported to significantly induce IL-10 production by immature DCs and Th2-type responses *in vitro* (Sun et al., 2012b). Furthermore, rSj16 has been shown to increase CD4^+^CD25^+^ regulatory T cells in splenic cells (Sun et al., 2012a). Recently, animal experiments confirmed that rSj16 protects against DSS-induced colitis by inhibiting the peroxisome proliferator-activated receptor α (PPAR- α) signaling pathway (Wang et al., 2017).

## Sm16

Sm16, a 16.8-kDa protein secreted by the parasite *S. mansoni* (cercariae and schistosomula) during skin penetration, has been reported to have anti-inflammatory and immunomodulatory activities (Ramaswamy et al., 1994; Kalyanasundaram et al., 1995). *In vitro* studies have shown that Sm16 downregulates ICAM-1 expression on endothelial cells, prevents lymphoproliferation, and suppresses IL-1a expression in human keratinocytes (Kalyanasundaram et al., 1995; Ramaswamy et al., 1998). Moreover, Sm16 can prevent LPS-induced neutrophil infiltration in the dermis (Rao and Ramaswamy, 2000). Sm16 potently inhibits the cytokine response to the Toll-like receptor ligands LPS and polyinosinic:polycytidylic acid, and it also inhibits the degradation of the IRAK1 signaling protein in LPS-stimulated monocytes (Brannstrom et al., 2009). Additionally, Sm16 may contribute to limit dermal inflammation after percutaneous infection with *S. mansoni* cercariae (Sanin and Mountford, 2015).

## Discussion

During the long history of the co-evolution of humans and parasites, parasites have adopted a parasitic lifecycle and gradually become exquisitely well-adapted to the host immune system, and they can live in the host for a long time by modulating the host immune response. Epidemiological investigation, animal models, and several clinical trials indicate that infections involving some parasites, especially helminths such as *S. mansoni, S. japonicum, Trichuris suis, Clonorchis sinensis, H. diminuta*, and *Ascaris lumbricoides*, can prevent or treat autoimmune and atopic diseases to a certain extent. There is growing interest in identifying the important parasite-derived immunomodulation molecules for use as autoimmune and atopic disease treatments, because directly using parasites as treatments may cause parasitic diseases and it would be difficult to make the treatments widely accepted by patients.

Parasite-derived immunomodulation molecules have different mechanisms of hampering the host immune response to create a tolerant environment not only to ensure their own survival but also to protect the host against excessive inflammation by reducing the immune response and inhibiting the progression of atopic diseases. They can exert their immunoregulatory functions by modulating cells of both the innate and adaptive immune systems. Generally, most parasites can promote a typical Th2 response and modulate Th1/Th17 differentiation, leading to a significant increase in the secretion of Th2-related cytokines such as IL-4, IL-5, IL-9, IL-10, IL-13, and IgE, and a decrease the secretion of Th1/Th17-related cytokines such as IL-6, IL-12, TNF-α, IFN-r, and IL-17 (Pulendran et al., 2010). For example, ES-62 derived from *A. viteae* has been discovered to promote a TH2 response by interacting with TLR4, modulate lymphocyte activation by modulating BCR and TCR, block mast cell degranulation (leading to the release of pro-inflammatory cytokines such as prostaglandins and leukotrienes), and modulate complement activation to protect against atopic diseases. ES-62 has been used to treat rheumatoid arthritis, systemic lupus erythematosus, ovalbumin-induced airway hypersensitivity, oxazolone-induced contact sensitivity, and so on (Lumb et al., 2017).

Parasite-derived immunomodulation molecules can evoke a regulatory or tolerogenic phenotype among immune cells including DCs, B cells, T cells, and macrophages. Helminth proteins can influence DC differentiation toward a tolerogenic phenotype by modulating the DC signaling pathway. These kinds of phenotypes, such as M2 macrophages and tolerogenic DCs, can shift a Th1/Th17 response to a Th2 response, and they have been shown to be essential for the prevention of autoimmunity (Versini et al., 2015). Some proteins also influence the innate immune cells such as ILCs. ILCs play a role in host-initiated protective immunity against helminths and are able to initiate allergies (Spits et al., 2013; Versini et al., 2015). In addition to direct regulation of the immune system, parasite-derived proteins can also indirectly regulate the immune system by modulating the bacterial composition of the gut microflora (Walk et al., 2010; Lee et al., 2014; Zaiss et al., 2015).

However, owing to the immunogenicity of parasite-derived molecules, these potential treatments may be harmful to some people. Analogs of parasite-derived molecules have been designed to function as immune modulators that have less or even no immunogenicity. For example, SMAs have been designed according to the structure of PC, and they have similar functions to PC but no immunogenicity. One of the SMAs, 11a, has been shown to inhibit the progression of collagen-induced arthritis in a murine model.

As the incidence and harmfulness of allergies and autoimmunity have been rising all over the world, the design of new drugs to prevent and treat these diseases should not be delayed. Parasites have long parasitized the human body and have lived in peace with their hosts by modulating the human immune response. The administration of parasite-derived molecules is considered more effective and safer to humans than other immunomodulatory drugs. However, research on parasite-derived molecules is just the beginning. Most experiments to investigate the functions of parasite-derived proteins, in terms of their effects on atopic diseases, have involved animal models. Thus, production quality standards and effective dosages of the parasite-derived proteins need to be confirmed. In addition, the precise mechanisms of these key proteins need to be explored further. There are still insufficient studies on the administration of the molecules among humans. From parasites to pills, there is still a long way to go.

## Author Contributions

This manuscript was designed by XS, drafted by ZW, LW, and YT, and revised by XS. All authors participated in critical revision of the manuscript and approved the final manuscript.

## Conflict of Interest Statement

The authors declare that the research was conducted in the absence of any commercial or financial relationships that could be construed as a potential conflict of interest.
